# Supplementary material for: Methyl-CpG-binding protein 2 reads histone methylation via an aromatic cage to regulate gene expression and chromatin association
Source: J Biol Chem. 2025 Dec 29;302(2):111115. doi: 10.1016/j.jbc.2025.111115 (PMC12857291; doi:10.1016/j.jbc.2025.111115)
Supplement: Supporting Information [file mmc1.pdf]

Supporting Information

Methyl-CpG binding protein 2 reads histone methylation *via* an aromatic cage to regulate gene expression and chromatin association

Jyotirmayee Padhan[1] and Babu Sudhamalla[1,*]

[1]Department of Biological Sciences, Indian Institute of Science Education and Research Kolkata, Mohanpur, West Bengal 741246, India.

[*]Correspondence: s.babu@iiserkol.ac.in

ORCID: Babu Sudhamalla: 0000-0002-6610-1424.

Table of Contents

1. Supplementary Tables S2
2. Supplementary Figures S3

Supplementary Table S1. List of the forward primers designed for site-directed mutagenesis. Reverse primers used are the reverse-complement to the given forward primers.

MeCP2-MBD Mutants	Primer Sequence
W104A	CCCACCCTGCCTGAAGGCGCGACACGGAAGCTTAAG
F132A	CCCCAGGGAAAAGCCGCGCGCTCTAAAGTGGAGTTGATTG
Y141A	GTGGAGTTGATTGCGGCGTTCGAAAAGGTAGGCGACAC
F142A	GTGGAGTTGATTGCGTACGCGGAAAAGGTAGGCGACAC
F155A	CTGGACCCTAATGATGCGGACTTCACGGTAACTGGG
R133C	CCCCAGGGAAAAGCCTTTTGCTCTAAAGTGGAGTTGATTGCG

Supplementary Table S2. List of primers used for the ChIP-qPCR.

**ChIP-qPCR Primers**		
**Gene**	**Forward**	**Reverse**
DNMT1	CAAAAGGGGAACCTTGTTCA	CCTGGGAGGAAGAAATAGGG
SIRT1	TAGACGCAACAGCCTCCG	GGCTGCGGGAGATTTAAACC
ICAM1	ATTGTCCGGGAAACTGGACG	ACAACAGGCGGTGAGGATTG
HIPK3	CCACTACCCCTCGCCCTA	CTGAGAGGAAACGGCGAAAC
DKK1	CGGTTCTCAATTCCAACGCT	CCCCTCTCACCTGGTAGTTG
ILF3	AGGCAGCTACTCCTACTCGAA	CACCACTTGTCCTCCTCCTAA
ICAM3	CATGGTCCAGTGGGAAAGGT	ATAGGCTTGTACGCCATCCC
KDM1A	AAACCCGAAAGTCCCTGGAG	GCAGCAAAGAACGTGTAGCT
ZNF713	AAAACAGACCCGGGAAAGCT	GACAGCCTCCCCGCAATG
HDAC1	ACTACTACGACGGTGAGCAC	CCTCCTCTCCGAGCCTCT

Supplementary Table S3: List of primers used for the qRT-PCR.

**qRT-PCR Primers**		
**Gene**	**Forward**	**Reverse**
DNMT1	CCTAGCCCCAGGATTACAAGG	ACTCATCCGATTTGGCTCTTTC
SIRT1	TAGCCTTGTCAGATAAGGAAGGA	ACAGCTTCACAGTCAACTTTGT
ICAM1	ATGCCCAGACATCTGTGTCC	GGGGTCTCTATGCCCAACAA
HIPK3	TCACAAGTCTTGGTCTACCCA	CACATAGGTCCGTGGATAGTTTC
DKK1	CCTTGAACTCGGTTCTCAATTCC	CAATGGTCTGGTACTTATTCCCG
ILF3	AGCATTCTTCCGTTTATCCAACA	GCTCGTCTATCCAGTCGGAC
KDM1A	TGACCGGATGACTTCTCAAGA	GTTGGAGAGTAGCCTCAAATGTC
HDAC1	CGCCCTCACAAAGCCAATG	CTGCTTGCTGTACTCCGACA

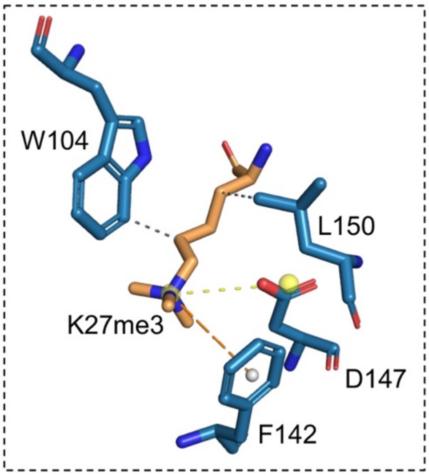

Supplementary Figure S1. Close-up view of the H3K27me3 peptide bound to the MeCP2-MBD, highlighting key protein-ligand contacts: hydrophobic interactions with W104 and L150, a cation–π interaction involving F142, and a salt-bridge interaction with D147.

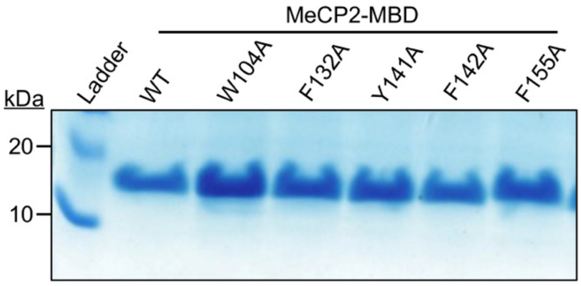

Supplementary Figure S2. Coomassie stained SDS-PAGE gel showing the overexpression of MeCP2-MBD wild-type and its mutants in BL21 Star (DE3) *E. coli* cells.

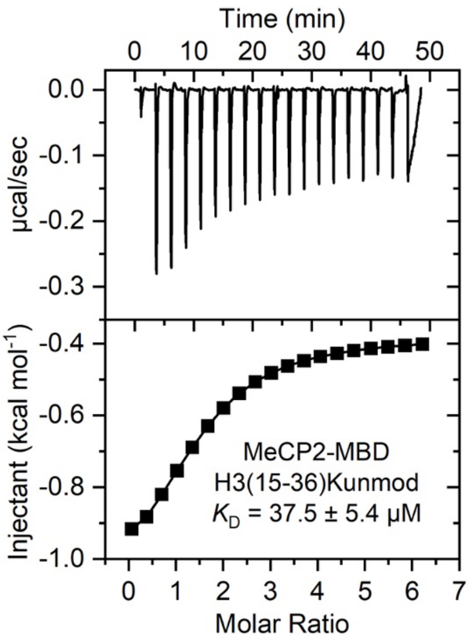

Supplementary Figure S3. Exothermic ITC plots showing binding of MeCP2-MBD domain to H3(15-36) unmodified peptide. The calculated binding constants are indicated.

HPLC REPORT

Sample:	Pep-685 APRKQLATKAARKSAPATGGVK		Analyzed date: 21-03-2023
Analyst:	Dr.AR-SBio		
Column:	4.6×250mm,Sinochrom ODS-BP 5μm		
Solvent A	A: 0.1% Trifluoroacetic Acid in 100% Acetonitrile		
Solvent B	B: 0.1% Trifluoroacetic Acid in 100% Water		
Gradient:		A	B
	0.0min	7%	93%
	25.0min	32%	68%
	25.1min	100%	0%
	30.0min		Stop
Volume:	5μl		
Wavelength:	220nm		
Flow rate:	1.0ml/min		

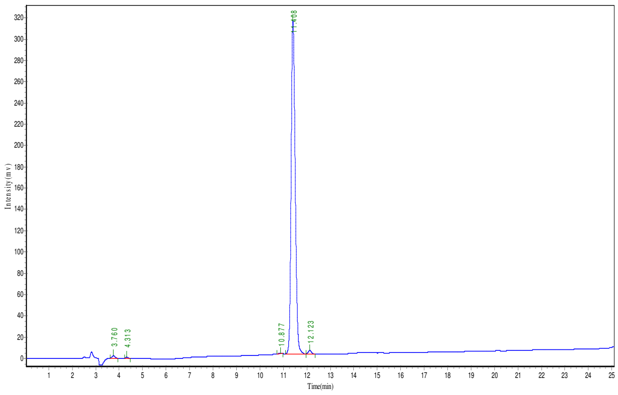

Peak	Time	Height	Area	Conc.
1	3.760	2243.948	19277.100	0.5076
2	4.313	1043.928	6693.750	0.1763
3	10.877	830.575	7119.000	0.1875
4	11.408	313463.625	3725986.500	98.1142
5	12.123	3722.384	38524.609	1.0144
Total				100.000

Supplementary Figure S4. HPLC purity trace for the histone H3(15-36) peptide.

MASS SPECTROMETRY REPORT

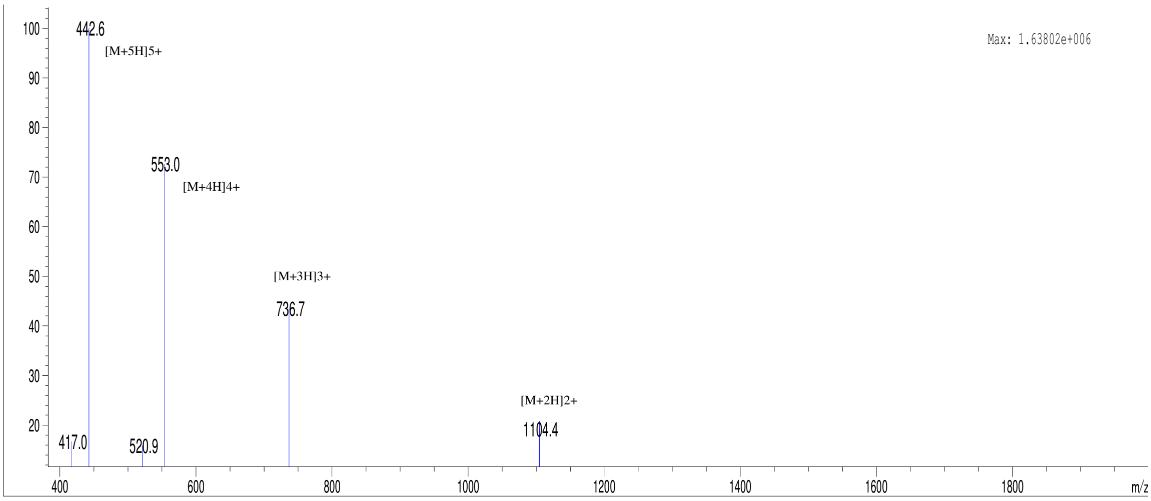

Sample Description		Instrument	Agilent-6125B		
Analyzed date:	22-03-2023	Probe:	ESI	Probe Bias:	+4.5kv
Analyst:	Dr.AR-SBio	Nebulizer Gas Flow:	1.5L/min	Detector:	1.5kv
Sample:	Pep-685 APRKQLATKAARKSAPATGGVK	CDL:	-20.0v	T. Flow:	0.2ml/min
M.W.:	2207.57	CDL Temp.:	250 °C	B. Conc.:	50%H2O/50%ACN

Supplementary Figure S5. MS spectra for the histone H3(15-36) peptide.

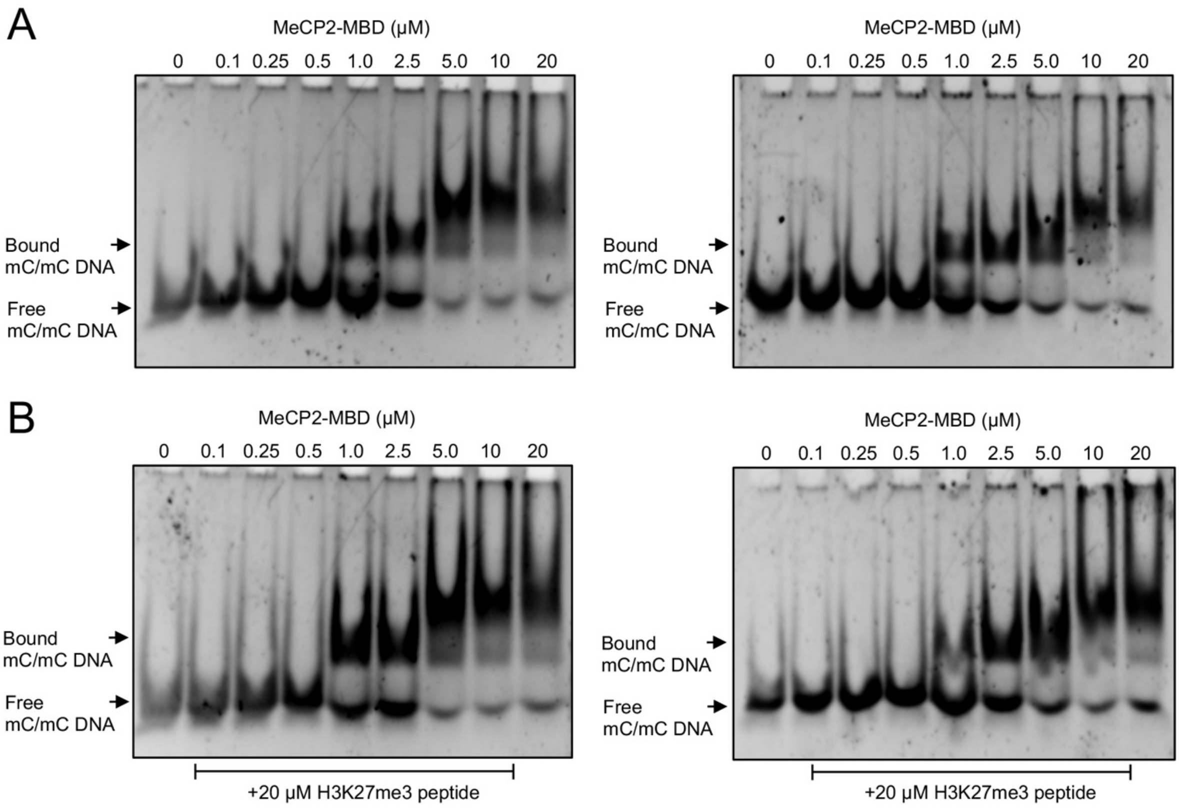

Supplementary Figure S6. EMSA titrations of the MeCP2-MBD with methylated DNA in the absence and presence of H3K27me3 peptide.

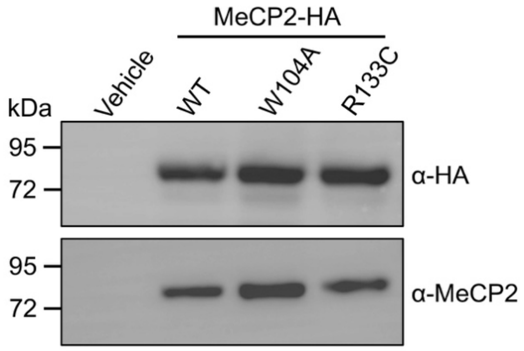

Supplementary Figure S7. Western blot analysis showing the expression of HA-MeCP2-WT and its mutants in HEK293T cells.

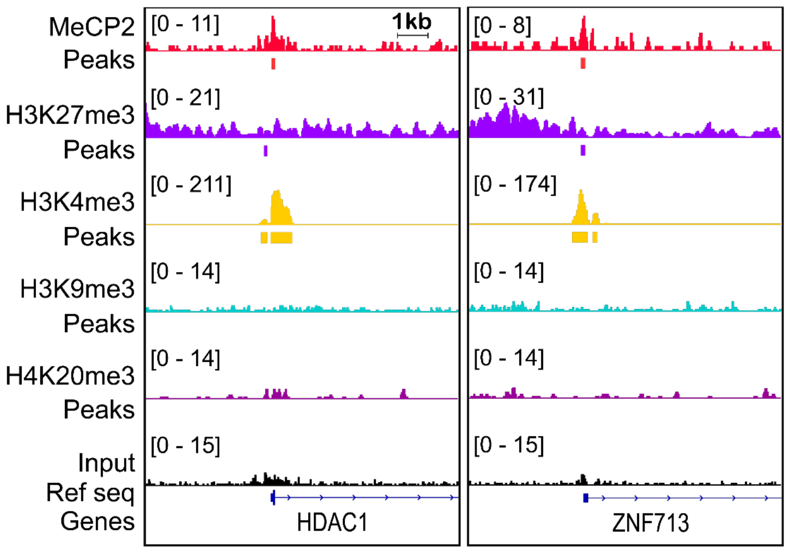

Supplementary Figure S8. IGV snapshots showing the co-localization of MeCP2 and different histone methylation marks at the promoters of MeCP2 target genes.

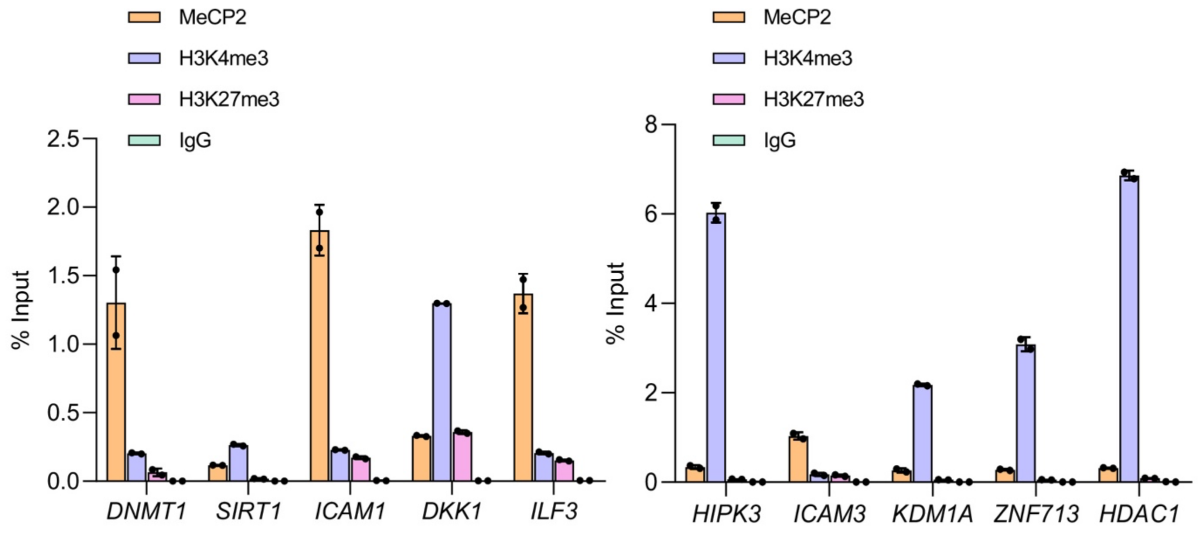

Supplementary Figure S9. ChIP-qPCR analysis of immunoprecipitated DNA to validate the presence of MeCP2 and histone marks, H3K4me3 and H3K27me3 in the indicated genes. Data are presented as mean ± SD (n = 2).

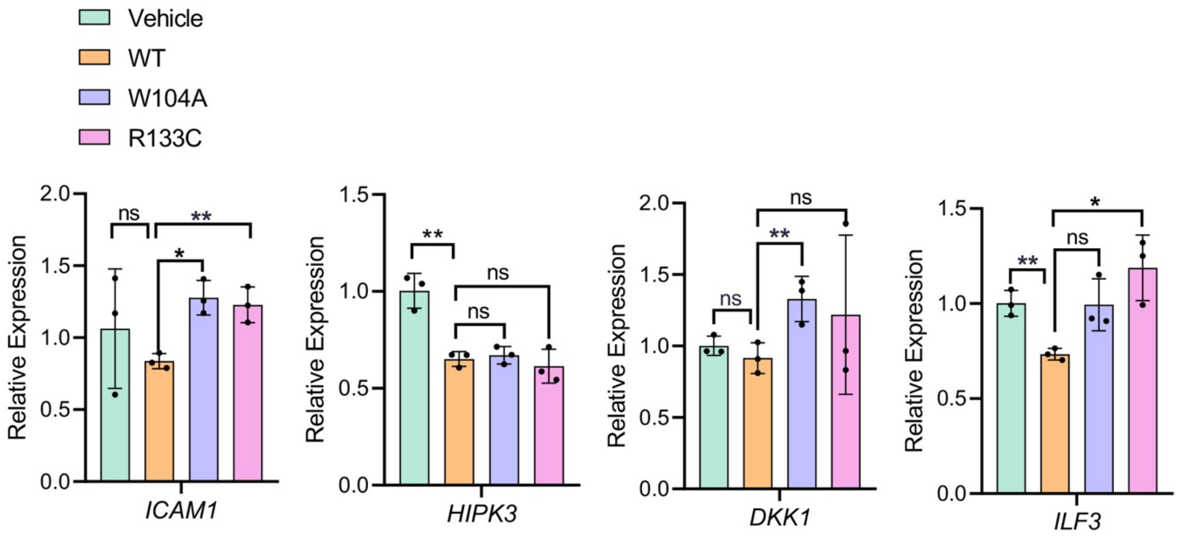

Supplementary Figure S10. qRT-PCR analysis of MeCP2 target genes in the presence of MeCP2-WT and its mutants W104A, and R133C. Data are presented as mean ± SD (n = 3). Statistical significance was determined using Student's t-test (*$p \leq 0.05$; **$p \leq 0.01$; ***$p \leq 0.001$; ns, not significant).

HPLC REPORT

Sample:	Pep-430 APRKQLATKAARK(me3)SAPATGGVK			Analyzed date: 30-08-2022
Analyst:	Dr.RS-SBio			
Column:	Gemini-NX 5μ C18 110A, 4.6*250mm			
Solvent A	A: 0.1% Trifluoroacetic Acid in 100% Acetonitrile			
Solvent B	B: 0.1% Trifluoroacetic Acid in 100% Water			
Gradient:		A	B	
	0.0min	5%	95%	
	25.0min	30%	70%	
	25.1min	100%	0%	
	30.0min		Stop	
Volume:	20μl			
Wavelength:	220nm			
Flow rate:	1.0ml/min			

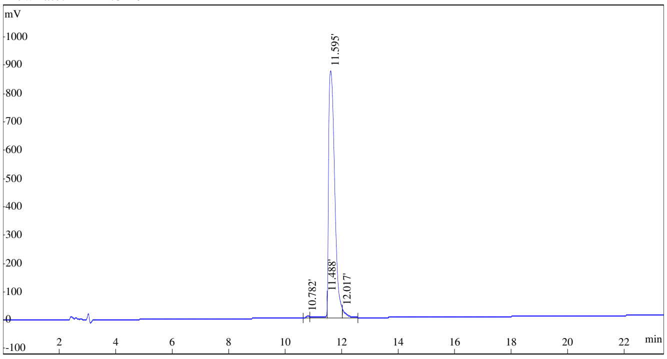

Rank	Time	Conc.	Area	Height
1	10.782	0.3655	46612	5840
2	11.488	1.1730	149603	76650
3	11.595	96.6699	12328976	868968
4	12.017	1.7916	228489	27967
Total		100	12753680	979425

Supplementary Figure S11. HPLC purity trace for the H3K27me3 (15-36) peptide.

MASS SPECTROMETRY REPORT

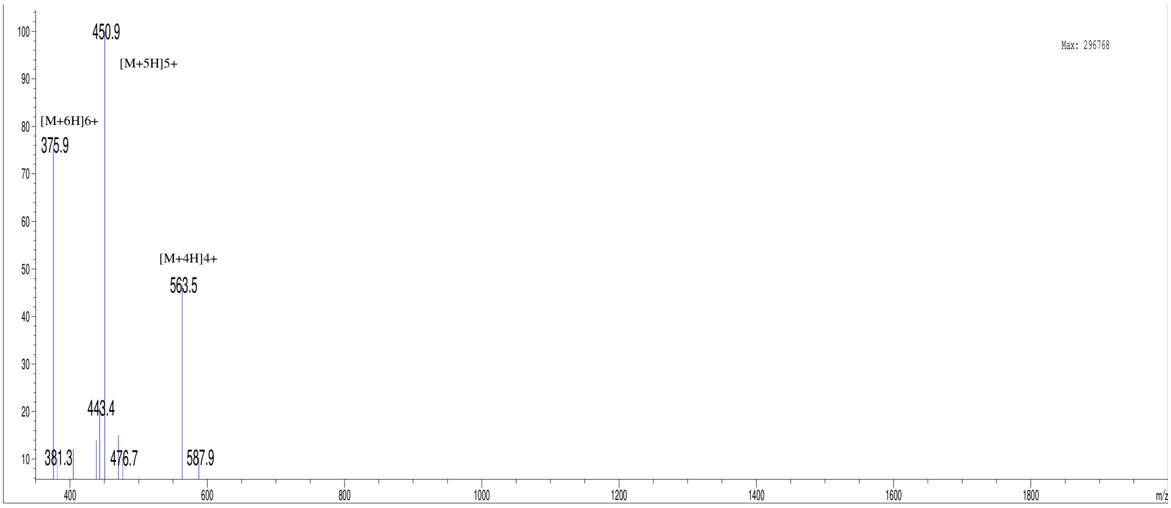

Sample Description		Instrument	Agilent-6125B		
Analyzed date:	31-08-2022	Probe:	ESI	Probe Bias:	+4.5kv
Analyst:	Dr.AR-SBio	Nebulizer Gas Flow:	1.5L/min	Detector:	1.5kv
Sample:	Pep-430 APRKQLATKAARK(me3)SAPATGGVK	CDL:	-20.0v	T. Flow:	0.2ml/min
M.W.:	2249.57	CDL Temp.:	250 °C	B. Conc.:	50%H2O/50%ACN

Supplementary Figure S12. MS spectra for the H3K27me3 (15-36) peptide.
